# Effect of population screening for type 2 diabetes and cardiovascular risk factors on mortality rate and cardiovascular events: a controlled trial among 1,912,392 Danish adults

**DOI:** 10.1007/s00125-017-4323-2

**Published:** 2017-08-23

**Authors:** Rebecca K. Simmons, Simon J. Griffin, Daniel R. Witte, Knut Borch-Johnsen, Torsten Lauritzen, Annelli Sandbæk

**Affiliations:** 1 MRC Epidemiology Unit, University of Cambridge School of Clinical Medicine, Box 285, Institute of Metabolic Science, Cambridge Biomedical Campus, Cambridge, CB2 0QQ UK; 2 Steno Diabetes Center, Gentofte, Denmark; 3 Department of Public Health, Section of General Practice, Aarhus University, Aarhus, Denmark; 4 Danish Diabetes Academy, Odense University Hospital, Odense, Denmark; 5 Aarhus Institute of Advanced Studies, Aarhus, Denmark; 6 Primary Care Unit, Institute of Public Health, University of Cambridge, Cambridge, UK; 7 Department of Public Health, Section of Epidemiology, Aarhus University, Aarhus, Denmark; 8 Holbæk Hospital, Holbæk, Denmark

**Keywords:** Cardiovascular disease, General practice, Mortality rate, Population, Screening, Type 2 diabetes

## Abstract

**Aims/hypothesis:**

Health check programmes for chronic disease have been introduced in a number of countries. However, there are few trials assessing the benefits and harms of these screening programmes at the population level. In a post hoc analysis, we evaluated the effect of population-based screening for type 2 diabetes and cardiovascular risk factors on mortality rates and cardiovascular events.

**Methods:**

This register-based, non-randomised, controlled trial included men and women aged 40–69 years without known diabetes who were registered with a general practice in Denmark (*n* = 1,912,392). Between 2001 and 2006, 153,107 individuals registered with 181 practices participating in the Anglo–Danish–Dutch Study of Intensive Treatment in People with Screen-Detected Diabetes in Primary Care (ADDITION)-Denmark study were sent a diabetes risk score questionnaire. Individuals at moderate-to-high risk were invited to visit their GP for assessment of diabetes status and cardiovascular risk (screening group). The 1,759,285 individuals registered with all other general practices in Denmark constituted the retrospectively constructed no-screening (control) group. Outcomes were mortality rate and cardiovascular events (cardiovascular disease death, non-fatal ischaemic heart disease or stroke). The analysis was performed according to the intention-to-screen principle.

**Results:**

Among the screening group, 27,177 (18%) individuals attended for assessment of diabetes status and cardiovascular risk. Of these, 1,533 were diagnosed with diabetes. During a median follow-up of 9.5 years, there were 11,826 deaths in the screening group and 141,719 in the no-screening group (HR 0.99 [95% CI 0.96, 1.02], *p* = 0.66). There were 17,941 cardiovascular events in the screening group and 208,476 in the no-screening group (HR 0.99 [0.96, 1.02], *p* = 0.49).

**Conclusions/interpretation:**

A population-based stepwise screening programme for type 2 diabetes and cardiovascular risk factors among all middle-aged adults in Denmark was not associated with a reduction in rate of mortality or cardiovascular events between 2001 and 2012.

**Electronic supplementary material:**

The online version of this article (doi:10.1007/s00125-017-4323-2) contains peer-reviewed but unedited supplementary material, which is available to authorised users.

## Introduction

As governments seek to apply the principles of prevention to chronic disease, health check programmes have been proposed or introduced in a number of countries, including the UK and the USA [1, 2]. These usually include assessment and management of risk factors for chronic disease, most of which are related to cardiovascular disease (CVD). Modelling studies suggest that screening for diabetes and cardiovascular risk assessment might be both effective and cost-effective; however, these studies rely on a number of assumptions [3–7]. There are relatively few trials assessing the benefits and harms of screening at the population level [8, 9]. A Cochrane review of randomised trials comparing health checks with no health checks in adult populations found that they did not reduce morbidity, all-cause mortality or cardiovascular-related mortality rates, although the number of new diagnoses increased [10]. The review included data from a number of historical cohorts that were initiated before the widespread introduction of effective treatments such as statins. More recent studies examining the impact of systematic population-wide screening have shown mixed results. INTER-99 reported no effect and the Västerbotten Intervention Programme, which combined screening with a wider public health promotion programme, reported mortality rate reductions [9, 11].

Given the limited evidence of the impact of population-based screening programmes, it is important to explore whether health checks might have different impacts in contemporary populations. It is also critical to evaluate the impact of screening on overall population mortality rates rather than simply disease-specific mortality or disease event rates to quantify overall benefits and harms at the population level [12]. Between 2001 and 2006, a population-based cardiovascular risk assessment and diabetes screening programme was introduced in five Danish counties as part of the Anglo–Danish–Dutch Study of Intensive Treatment in People with Screen-Detected Diabetes in Primary Care (ADDITION) study [13]. The Danish national registration system enables a post hoc analysis of the rates of mortality and cardiovascular events in individuals who were invited to take part in the ADDITION-Denmark screening programme compared with individuals who were not invited during the same time period.

## Methods

ADDITION-Denmark consists of two phases: a pragmatic screening programme; and a cluster-randomised trial comparing the effects of intensive multifactorial therapy with routine care among individuals with screen-detected type 2 diabetes [13, 14]. We report results from a post hoc analysis of data from the screening phase of the study in conjunction with outcome data from Danish national registers. Ethical approval for the ADDITION-Denmark study was granted by a local scientific committee (no: 20000183). As this was a registry-based study using anonymised data, participants did not give informed consent. This approach was approved by the Danish Data Protection Agency and the Danish Health and Medicine Authority.

### Intervention

We performed a population-based stepwise screening programme among people aged 40–69 years without known diabetes between 2001 and 2006. Full details have been published previously [13–15]. All general practices in five out of 16 counties in Denmark (Copenhagen, Aarhus, Ringkøbing, Ribe and South Jutland) were invited to take part in ADDITION-Denmark (*n* = 744); 209 (28.1%) accepted (county-specific proportions ranged from 21 to 37%). Eligible individuals aged 40–69 years without diagnosed diabetes who were registered with 181 general practices that were part of the study were sent a diabetes risk score questionnaire [14, 15]. Individuals who scored ≥5 points (maximum 15 points) were invited to visit their general practitioner (GP) for a diabetes test and a cardiovascular risk assessment. The risk score was developed using the Danish Inter99 population [15]. The sensitivity and specificity for predicting undiagnosed diabetes are 68.9–77.0% and 68.8–78%, respectively, using four different cut-off points [15]. External validation using the chosen cut-off point of ≥5 was completed using data from 1028 individuals in a pilot for the ADDITION study and revealed a sensitivity of 76.0% (95% CI 58.3, 90.3) and specificity of 72.2% (69.3, 75.1) for diabetes. In Aarhus and Copenhagen counties, 35 practices also completed opportunistic screening, in which individuals were asked to complete the risk score questionnaire when attending the practice for other reasons. Participants who attended a screening appointment underwent measurement of height, weight, BP, random blood glucose (RBG), total cholesterol and HbA_1c_. Participants also answered a question about their smoking status. GPs were encouraged to calculate the European Heart SCORE [16] during the appointment, to inform individuals about their score and provide appropriate advice to those at high risk. Individuals with an RBG ≥5.5 mmol/l or HbA_1c_ ≥ 5.8% (40 mmol/mol) were invited to return to the practice for a fasting capillary blood glucose (FBG) test. An OGTT was performed at the same consultation if FBG was 5.6–6.1 mmol/l and/or HbA_1c_ ≥ 5.8%. GPs were notified of all results. The WHO 1999 criteria were used to diagnose diabetes [17], including the requirement for a confirmatory test on another day.

Participants diagnosed with type 2 diabetes were subsequently managed according to the treatment regimen to which their practice had been allocated: routine care or intensive treatment [18]. For individuals found to have impaired fasting glucose (IFG) or impaired glucose tolerance (IGT) [17], and/or a high CVD risk (SCORE >5 points), practitioners were encouraged to manage cardiovascular risk factors according to national guidelines [19], including annual cardiovascular risk assessment and diabetes tests.

**Fig. 1:**
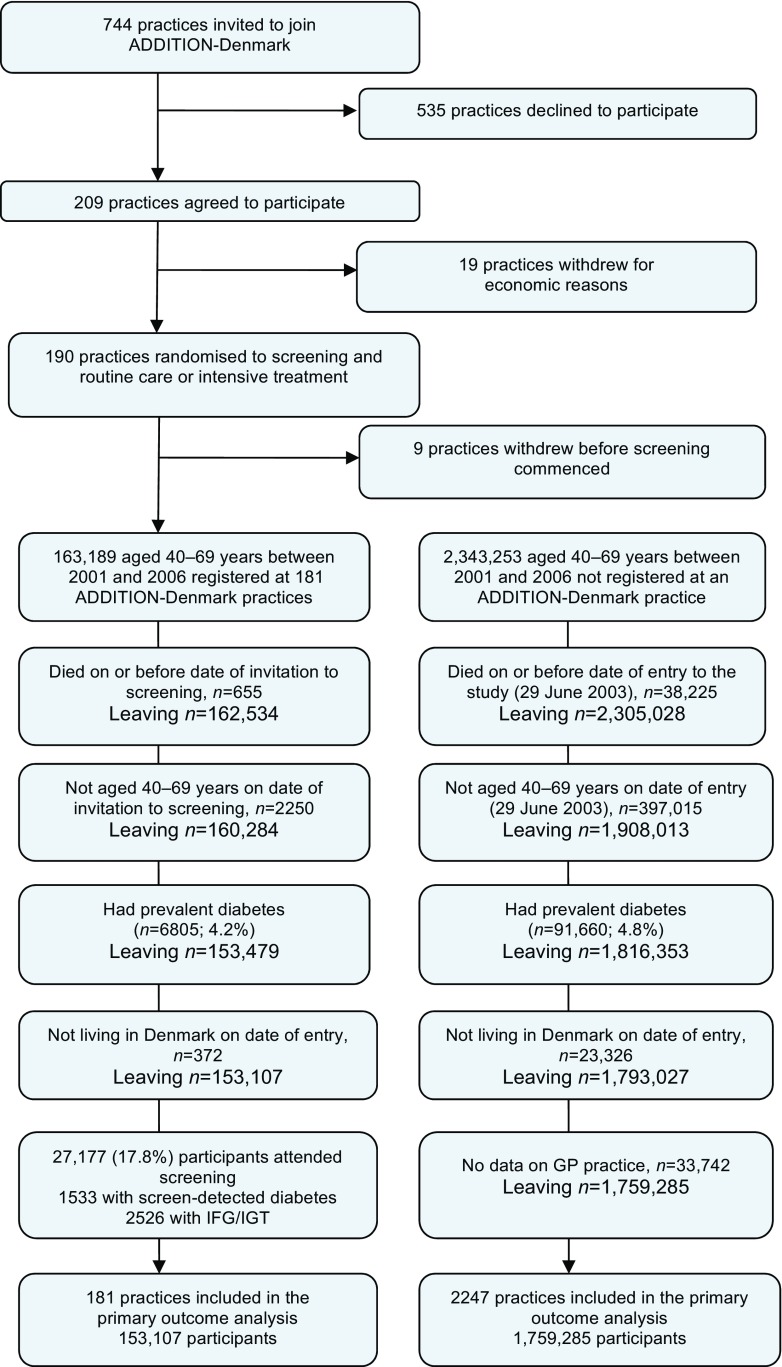
ADDITION-Denmark screening flow

In order to assess the impact of invitation to screening on population mortality rates, all individuals in the original ADDITION-Denmark sampling frame (*n* = 153,107), including those who did not attend for screening, were identified on the Danish National Registry system (the screening group). Using the same registry, we also identified all individuals aged 40–69 years without known diabetes who, between 2001 and 2006, were registered with general practices that were not invited or who declined to take part in ADDITION-Denmark (*n* = 1,759,285) (the no-screening group) (Fig. 1). We linked information about these individuals to other Danish registers using unique civil registration numbers. We retrieved information on age, sex, education, region, immigration/emigration, citizenship, redeemed cardioprotective medication and chronic disease (ischaemic heart disease [IHD], stroke, cancer). Education was categorised according to UNESCO’s International Standard Classification of Education [20]. We grouped data on citizenship into European and non-European citizens as a proxy for ethnicity.

### Outcomes

Participants were followed for a median of 9.5 years to 31 December 2012, when national registers were searched for information on vital status and incident CVD events. For death, the primary outcome was all-cause mortality rate (based on underlying cause of death). Secondary outcomes were cardiovascular-, cancer- and diabetes-related mortality rates. Cause-specific deaths were coded blind to study group using ICD-10 codes (www.who.int/classifications/icd/en/; electronic supplementary material (ESM) Table 1). For CVD, the primary outcome was a composite of first event of cardiovascular death, non-fatal IHD (ICD-10 codes I20–I25 and I46) or non-fatal stroke (ICD-10 code I6*). Data on incident CVD events was gathered from the National Patient Registry, which records all inpatient and outpatient hospitalisations in Denmark.

### Statistical analysis

Analysis was by intention-to-screen at the population level comparing outcomes in people registered at screening practices with those registered at no-screening practices. All eligible participants were considered irrespective of their participation in the screening programme. Baseline characteristics were summarised separately in the screening and no-screening (control) groups using the unpaired *t* test for continuous data and *χ* ^2^ test for categorical data. Date of entry to the study for individuals in the screening practices was the date of invitation to screening. Date of entry for the no-screening (control) practices was the mid-point (29 June 2003) of the randomisation period of ADDITION-Denmark practices (2001–2006). Individuals were censored on the date of first event, date of emigration or 31 December 2012, whichever was earliest. HRs comparing mortality rates and CVD outcomes between the screening and no-screening groups were estimated using a Cox proportional hazards regression model. As allocation to screening in ADDITION-Denmark was at the practice level, robust standard errors were calculated that take into account the two-level structure of the data (individuals clustered within practices) [21] and any potential correlation between individuals within practices. We adjusted for age, sex, education and prevalent chronic disease (IHD, stroke, cancer). To further account for differences in social structure, we stratified the baseline hazards by county. We also compared outcomes between screening attenders with the no-screening group. Attendance was defined as attending the initial consultation for diabetes testing and CVD risk assessment. To assess possible bias from unknown antecedent disease, sensitivity analyses were conducted omitting deaths from both groups occurring within 1 year of randomisation (*n* = 12,375). We also re-ran the Cox model comparing mortality outcomes after excluding individuals affiliated to general practices undertaking opportunistic screening. We calculated the proportion of participants who redeemed prescriptions for lipid-lowering, anti-hypertensive and glucose-lowering medication from 2000 to 2008 in both groups. All analyses were completed using Stata version 14.1 (STATA, College Station, Texas, USA).

## Results

The screening and control groups were well balanced for age, sex and citizenship at baseline (Table 1). Compared with the control group, a larger proportion of the screening group (330,096 [19.3%] vs 34,648 [23.1%]) had received >15 years of education. Slightly higher proportions of the control group had experienced IHD, stroke or cancer compared with the screening group.

**Table 1:** Baseline characteristics of participants in the screening and no-screening groups (2001–2006)

Characteristic	Screening group (*n* = 153,107)	No-screening (control) group (*n* = 1,759,285)
Mean age (SD), years	53.6 (8.1)	53.4 (8.2)
Male sex, *n* (%)	75,569 (49.4)	875,241 (49.8)
Years of education, *n* (%)^a^		
0–10	46,232 (30.8)	571,727 (33.4)
10–15	69,025 (46.1)	808,851 (47.3)
> 15	34,648 (23.1)	330,096 (19.3)
European citizenship, *n* (%)^a^	151,937 (99.3)	1,738,603 (99.0)
Previous IHD^b^_,_ *n* (%)	4989 (3.3)	63,734 (3.6)
Previous stroke^b^_,_ *n* (%)	2313 (1.5)	29,023 (1.7)
Previous cancer^b^_,_ *n* (%)	15,210 (9.9)	179,000 (10.2)

^a^Totals do not match denominator owing to missing data

^b^Data taken from the National Patient Registry; data available from 1994 onwards

Of 153,107 eligible people in the screening group, 27,177 (18%) attended their GP for a diabetes test and a cardiovascular risk assessment. Of these, 1533 participants (1% of those eligible for screening) were diagnosed with diabetes and 2526 (1.6%) had IFG/IGT. Of all individuals who attended screening for ADDITION-Denmark with complete data for calculating CVD risk, 9693/20,223 (48%) had high CVD risk (European Heart SCORE ≥ 5 points). In total, 6855 (28%) of attenders with available data reported being a current smoker. There were 1,759,285 individuals in the no-screening control group.

### Effect of screening on mortality rate

Median duration of follow-up was 9.5 years (16,954,630 person-years). During follow-up, there were 11,826 deaths in the screening group (7.7%) and 141,719 in the no-screening control group (8.1%) (Table 2). The most common cause of death was cancer in the two groups (*n* = 67,694, 44.5%). Following adjustment, there was no significant difference in the all-cause mortality rate between the screening and the control groups (HR 0.99 [95% CI 0.96, 1.02], *p* = 0.66) (Table 2). Sensitivity analyses showed that the overall result was not affected by the exclusion of people who died within 1 year of the beginning of the study (*n* = 12,375; 0.99 [0.96, 1.02], *p* = 0.60) or of individuals affiliated to general practices undertaking opportunistic screening (1.00 [0.97, 1.03], *p* = 0.93). Compared with the no-screening group, attending for screening was associated with a 25% reduction in risk of death (0.75 [0.71, 0.78]).

**Table 2:** Incidence of all-cause and cardiovascular-, cancer- and diabetes-related mortality, and CVD events, by screening group (2001–2012)

Variable	Screening group (*n* = 153,107)			No-screening group (*n* = 1,759,285)			Adjusted HR^a^ (95% CI)
	Number of events	Person-years of follow-up	Rate per 1000 person-years (95% CI)	Number of events	Person-years of follow-up	Rate per 1000 person-years (95% CI)	
Mortality							
All-cause	11,826	1,425,981	8.29 (8.15, 8.44)	141,719	15,972,368	8.87 (8.83, 8.92)	0.99 (0.96, 1.02)
Cardiovascular-related	2291	1,425,981	1.61 (1.54, 1.67)	27,435	15,972,368	1.72 (1.70, 1.74)	1.02 (0.96, 1.08)
Cancer-related	5387	1,425,981	3.78 (3.68, 3.88)	62,307	15,972,368	3.90 (3.87, 3.93)	0.99 (0.96, 1.03)
Diabetes-related^b^	74	1,425,981	0.05 (0.04, 0.07)	904	15,972,368	0.06 (0.05, 0.06)	1.10 (0.84, 1.44)
Composite cardiovascular event^c^	17,941	1,352,463	13.28 (13.08, 13.47)	208,476	15,148,258	13.76 (13.70, 13.82)	0.99 (0.96, 1.02)

^a^HR was estimated with a Cox proportional hazards regression model. Robust SEs were calculated taking into account the two-level structure of the data and any potential correlation between individuals within practices. Models were adjusted for age, sex, education and prevalent chronic disease (IHD, stroke, cancer); baseline hazards were stratified by county

^b^Diabetes-related mortality includes any death with an ICD-10 code E10* to E14*

^c^First of CVD death, non-fatal IHD or non-fatal stroke

There was no significant difference between groups in cardiovascular mortality rate (HR 1.02 [95% CI 0.96, 1.08], *p* = 0.60) and cancer mortality rate (0.99 [0.96, 1.03], *p* = 0.69) (Table 2). Diabetes was listed anywhere on the death certificate for 981 individuals (77 in the screening group [0.7%] and 904 in the control group [0.6%]). There was no significant difference between groups in diabetes-related mortality rate (1.10 [0.84, 1.44], *p* = 0.51; Table 2).

### Effect of screening on CVD

Median duration of follow-up was 9.5 years (16,499,722 person-years). Following entry to the study, there were 17,941 first CVD events in the screening group (11.7%) and 208,476 in the no-screening group (11.8%) (HR 0.99 [95% CI 0.96, 1.02], *p* = 0.49) (Table 2). The composite CVD event included 12,552 CVD deaths, 138,809 non-fatal IHD and 75,057 non-fatal strokes.

### Cardioprotective medication

At baseline, the proportion of participants redeeming cardioprotective medication was similar in both groups (Table 3). Following commencement of the screening programme in 2001, larger numbers of people in the screening practices redeemed glucose-lowering medication compared with control practices (after excluding individuals with prevalent diabetes in each practice). This difference narrowed over time but was maintained until 2007, when both groups were prescribed similar levels of glucose-lowering medication (30,824 [1.81%] in the no-screening group and 2769 [1.87%] in the screening group). Similarly, larger numbers of people in the screening practices redeemed lipid-lowering prescriptions compared with the control practices. Similar proportions of participants in both groups redeemed prescriptions for anti-hypertensive medication throughout the follow-up period.

**Table 3:** Redeemed cardioprotective medication from 2000 to 2008

Year^a^	Glucose-lowering medication			Lipid-lowering medication			Anti-hypertensive medication		
	Screening	No-screening	*p* value	Screening	No-screening	*p* value	Screening	No-screening	*p* value
2000	14 (0.01)	215 (0.01)	0.29	3137 (2.1)	36,286 (2.1)	0.72	20,629 (13.5)	241,916 (13.8)	0.0025
2001	83 (0.05)	188 (0.01)	<0.0001	3921 (2.56)	45,443 (2.58)	0.63	23,097 (15.09)	268,441 (15.26)	0.086
2002	275 (0.18)	219 (0.01)	<0.0001	5402 (3.54)	59,709 (3.39)	0.0030	25,884 (16.95)	298,665 (16.98)	0.79
2003	666 (0.44)	3373 (0.19)	<0.0001	7836 (5.15)	82,590 (4.71)	<0.0001	29,087 (19.13)	333,731 (19.03)	0.34
2004	1103 (0.73)	9652 (0.55)	<0.0001	10,974 (7.26)	114,726 (6.59)	<0.0001	32,117 (21.24)	371,711 (21.34)	0.35
2005	1567 (1.04)	15,899 (0.92)	<0.0001	13,712 (9.12)	148,728 (8.60)	<0.0001	35,286 (23.47)	407,319 (23.55)	0.50
2006	2119 (1.42)	22,810 (1.33)	0.0036	17,116 (11.47)	191,917 (11.18)	0.0010	38,826 (26.01)	446,981 (26.05)	0.74
2007	2769 (1.87)	30,824 (1.81)	0.11	21,029 (14.20)	236,299 (13.89)	0.0011	42,207 (28.49)	485,665 (28.55)	0.66
2008	3490 (2.38)	40,117 (2.38)	0.93	25,034 (17.04)	284,675 (16.88)	0.12	45,454 (30.94)	522,555 (30.99)	0.69

Data are presented as *n* (%)

ATC codes: glucose-lowering medication (A*); lipid-lowering medication (C10*); anti-hypertensive medication (CO7*, CO8*, C09*)

^a^Denominator population based on participants who were alive on 1 January for each year

## Discussion

In this population-based sample of nearly two million middle-aged Danish adults, a single round of screening for type 2 diabetes and cardiovascular risk assessment was not associated with a reduction in the rates of all-cause mortality or cardiovascular events between 2001 and 2012. Similarly, rates of cardiovascular-, cancer- and diabetes-related mortality were not reduced by invitation to screening.

Modelling studies have previously suggested a benefit of population screening for diabetes and related risk factors for vascular disease [3–7]. This finding was supported by examination of the Ely cohort [22], in which individuals aged 40–65 years who were invited to diabetes screening every 5 years between 1990 and 1999 had a non-significant 21% lower all-cause mortality rate than individuals who were not invited to screening; however, this finding was not replicated in between 2000 and 2008.

A more recent screening and health promotion CVD intervention undertaken in Västerbotten County, Sweden, also showed a significant long-term reduction in all-cause and CVD mortality rates when compared with the general Swedish population [11]. This complex intervention was different from the ADDITION-Denmark study. The screening programme and health counselling offered to individuals was nested within a public health prevention programme targeting the wider collective determinants of diabetes and CVD. However, our findings mirror those of a pragmatic, parallel-group, cluster-randomised trial in 33 general practices in eastern England (ADDITION-Cambridge) [8], in which screening for type 2 diabetes in those at high risk did not result in a reduction in all-cause or cardiovascular- or diabetes-related mortality rates over 10 years. Our results also reaffirm a recent Cochrane review [10], which found no long-term impact of general health checks on mortality and morbidity rates following population screening.

There are many potential explanations for the lack of difference in mortality rates and cardiovascular events observed in this trial. The study was undertaken against a background of national interest about screening and early treatment for diabetes in Denmark. The effect of screening may therefore have been diluted by opportunistic screening in primary care and by continuing improvement in the detection and management of CVD risk factors. Only 27,177 (18%) of individuals in the screening group attended their general practice for testing which combined with the less than 100% sensitivity at each step of the screening programme might have contributed to the low yield of individuals found to have detectable disease (1% of the invited population) [14]. Although earlier detection may have benefitted those diagnosed with diabetes [18], the proportion was probably too small to affect population mortality rates. Furthermore, screening attendees are frequently those at lowest risk [23].

For cancers such as prostate, any effect of screening on population mortality rates is achieved largely through the effect of treatment in the lead time between detection by screening and clinical diagnosis [24]. By contrast, CVD risk factor screening identifies a significant proportion of the population that might benefit from health promotion and lifestyle change, which could also impact other health outcomes such as cancer. Unlike the ADDITION-Cambridge trial, the Danish screening programme included assessment of CVD risk factors alongside screening for undiagnosed prevalent diabetes. This is similar to recommendations in the UK NHS Health Checks Programme [1]. Danish GPs were encouraged to intervene in individuals with IFG/IGT and those with high CVD risk but without diagnosed diabetes. While small numbers of individuals were diagnosed with diabetes following screening in Denmark (1%), 1.6% were found with IFG/IGT and 48% with elevated cardiovascular risk (SCORE > 5 points), providing a larger at-risk group with the potential to benefit from interventions to prevent both diabetes and CVD [25, 26]. Indeed, larger proportions of people in the screening practices redeemed cardioprotective medication compared with the control practices during the screening phase of the study (2001–2006). However, these differences were small and were not sustained. Furthermore, there is evidence of sub-optimal treatment among individuals identified at high risk in health check programmes [27] and among those with screen-detected diabetes [18], which might have contributed to the lack of difference between groups. Indeed, follow-up of ADDITION-Denmark participants who did not have diabetes but had high CVD risk and dyslipidaemia showed that only 20% were started on lipid-lowering treatment following screening [28]. Of these, only 53% reached the treatment goal of total cholesterol <5.0 mmol/l and LDL-cholesterol <3 mmol/l within 1 year of starting treatment [29]. Among those with screen-detected diabetes, there was wide variation between general practices in prescription of lipid-lowering treatment [30]. As such, there is evidence of both under-treatment and considerable delay in starting treating among this high-risk population. Benefits to the population might have been increased by identification of non-attenders, targeting of screening to those at greatest risk, strategies to maximise uptake of screening (particularly among those at highest risk), repeated rounds of screening and optimal treatment of detected disease. Benefits might also be increased by introducing complex interventions that target collective determinants of chronic disease alongside individualised screening and treatment programmes. For example, the Västerbotten Intervention Programme aimed to raise public awareness of CVD risk factors and lifestyle behaviours by tailoring activities to the local community, including the introduction of ‘the green keyhole’ food labelling system denoting low fat and high fibre foods, development of healthy school lunches, production and distribution of health educational materials, and health information meetings [11]. Participation rates in Västerbotten were 48–67%. Our results underline the continuing uncertainty about the overall benefits of population screening for diabetes and CVD.

As with all screening programmes, positive health outcomes are associated with uptake. In common with findings from other population-based screening programmes, we observed the lowest mortality rates in attenders, probably due, at least in part, to healthy volunteer bias.

### Strengths and limitations

This very large non-randomised, controlled trial with 9.5 years of follow-up included all individuals aged 40–69 years without diagnosed diabetes in Denmark between 2001 and 2006. Outcome ascertainment was robust. The National Death Registry estimates 100% coverage of mortality based on death certificates. All-cause mortality is an all-inclusive measure that addresses both direct and indirect benefits and harms of screening, and puts disease-specific mortality rate reduction in the context of other competing risks [12]. We were able to ascertain which individuals were living in Denmark during the screening period and those who emigrated during follow-up. Deaths and CVD events were coded blind to study group. We used a validated risk score developed and evaluated in a Danish population to identify those at high risk of undiagnosed diabetes [15].

A limitation of our study includes the post hoc, non-randomised design, which means that we cannot eliminate the possibility of selection bias and residual confounding. Groups were well balanced for most baseline characteristics. However, there were higher levels of education and slightly lower levels of pre-existing chronic disease in the screening compared with the no-screening group. These differences would have tended to increase the apparent benefits of screening. In order to minimise the impact of these small baseline differences between study groups, we adjusted for age, sex, education and prevalent chronic disease at the individual level. We also accounted for clustering and stratified the baseline hazard function by county to allow for potential baseline differences in underlying determinants of disease by geographical region.

In terms of practice characteristics, data were not available with which to compare the screening and no-screening groups. The small differences at baseline between study groups suggest that practices participating in ADDITION-Demark served less deprived regions than the average Danish practice. The benefits of screening might be greater among more socioeconomically deprived communities in which the absolute disease risk is higher, although attendance for screening is likely to be lower [23]. The vast majority of participants were white European, the main ethnic group in Denmark, which also limits generalisability to other settings. We did not measure the potential psychological harms of screening for diabetes and related cardiovascular risk factors and subsequent treatment in ADDITION-Denmark. However, previous research suggests that such adverse effects are limited in both the short and longer term [31, 32].

While we were able to compare trends in redeemed cardioprotective medication to explore a potential effect of the screening programme, it would also have been useful to examine diet, physical activity and smoking behaviour. However, these data are not available for the entire Danish population. One-third of the individuals diagnosed with diabetes by screening in ADDITION-Denmark reported that they had stopped smoking by five-year follow-up. Furthermore the cohort lost an average of 2 kg in weight [18]. These behavioural responses further suggest that detection of diabetes by screening was associated with a positive impact among those found to have undiagnosed prevalent disease. Indeed, the results of this study must be placed in a wider context about the benefits of screening for those with detectable disease. There is growing evidence for the benefit of intensive treatment of risk factors early in the course of the disease [33]. Results from ADDITION-Europe*,* a cluster-randomised trial of intensive, target-driven management of screen-detected individuals, showed that those identified and treated earlier had a mortality rate that was similar to that reported for people of the same age without diabetes in the general population in Denmark [18]. The possibility also remains that screening for diabetes and CVD risk factors followed by multifactorial treatment may have effects on microvascular and other morbidity not evaluated in this study. Furthermore, in a separate paper comparing the mortality rate and cardiovascular outcomes in individuals with incident diabetes in the screened group with those from the unscreened group, we show significant benefits for those diagnosed with diabetes in the screening practices [34]. Thus, while screening for diabetes and CVD risk factors may not have an impact at the population level, it appears to have benefits for the small subgroup found to have undiagnosed prevalent diabetes.

In conclusion, invitation to one round of screening for type 2 diabetes and cardiovascular risk assessment among middle-aged adults in Danish general practice was not associated with a significant reduction in mortality rate or CVD events between 2001 and 2012. The benefits of population-based screening may be lower than expected and limited to individuals with detectable disease.

## Electronic supplementary material

ESM Table 1 (PDF 10 kb)

## Abbreviations

ADDITION: Anglo–Danish–Dutch study of intensive treatment in people with screen-detected diabetes in primary care
CVD: Cardiovascular disease
FBG: Fasting blood glucose
GP: General practitioner
IFG: Impaired fasting glucose
IGT: Impaired glucose tolerance
IHD: Ischaemic heart disease
RBG: Random blood glucose
