# Supplementary material for: Effect of population screening for type 2 diabetes and cardiovascular risk factors on mortality rate and cardiovascular events: a controlled trial among 1,912,392 Danish adults
Source: Diabetologia. 2017 Aug 23;60(11):2183–91. doi: 10.1007/s00125-017-4323-2 (PMC6086322; doi:10.1007/s00125-017-4323-2)
Supplement: Supplementary file 1 — (PDF 10 kb) [file 125_2017_4323_MOESM1_ESM.pdf]

ESM Table One Cause-specific mortality groupings by ICD-10 code

**Cause-specific mortality**	**ICD-10 code used**
Cardiovascular	F01*, I*
Cancer	C*, D0* to D4*,
Suicide / violence / accident	F1*, V*, W*, X*, Y*
Other	A*, B*, D5* to D8*, E*, F00*, F02* to F09*, F2* to F9*, G*, H*, J*, K*, L*, M*, N*, P*, Q*
Unknown	R*
**Diabetes-related mortality**	E10* to E14*
